# Non-Invasive Tests of Liver Fibrosis Help in Predicting the Development of Hepatocellular Carcinoma among Patients with NAFLD

**DOI:** 10.3390/jcm11092466

**Published:** 2022-04-27

**Authors:** Mònica Pons, Jesús Rivera-Esteban, Ramiro Manzano, Juan Bañares, María Bermúdez, Víctor Vargas, Maria Teresa Salcedo-Allende, Lluís Castells, Salvador Augustin, Beatriz Mínguez, Juan M. Pericàs

**Affiliations:** 1 Liver Unit, Vall d’Hebron University Hospital, 08035 Barcelona, Spain; jesus.rivera@vhir.org (J.R.-E.); ramiro.manzano@vhir.org (R.M.); juanbanares@gmail.com (J.B.); maria.bermudez@vhebron.net (M.B.); vvargas@vhebron.net (V.V.); llcastells@vhebron.net (L.C.); salva.agustin@gmail.com (S.A.); bminguez@vhebron.net (B.M.); 2 Vall d’Hebron Institut de Recerca (VHIR), Vall d’Hebron Barcelona Campus Hospitalari, 08035 Barcelona, Spain; 3 Faculty of Medicine, Universitat Autònoma de Barcelona, 08035 Barcelona, Spain; mtsalcedo@vhebron.net; 4 Centro de Investigación Biomédica en Red de Enfermedades Digestivas y Hepáticas (CIBERehd), 28029 Madrid, Spain; 5 Pathology Department, Vall d’Hebron University Hospital, 08035 Barcelona, Spain

**Keywords:** NAFLD, hepatocellular carcinoma, FIB-4, transient elastography

## Abstract

Background: The potential role of non-invasive tests (NITs) for liver fibrosis for hepatocellular carcinoma (HCC) prediction remains poorly known. Methods: Retrospective analysis of a NAFLD cohort from a single university hospital in Barcelona, Spain. Incidence rates and cumulative incidence for the overall cohort, as well as cirrhotic and non-cirrhotic patients were calculated. Logistic regression analyses were carried out to investigate risk factors of HCC. Results: From the entire cohort of 1040 patients, 996 patients (95.8%) were analyzed, in whom 35 cases of HCC were detected, of which 26 (72.4%) HCC incident cases were newly diagnosed during a median follow-up of 2.5 (1.9–3.6) years. Two-hundred and thirty-one (23.2%) were cirrhotic at baseline. With the exception of 2 (7.7%) cases of HCC, the rest were diagnosed in cirrhotic patients. Overall HCC cumulative incidence was 9.49 (95% CI 6.4–13.9) per 1000 person-years. The incidence rate for cirrhotic patients was 41.2 (95% CI 27.6–61.6) per 1000 person-years and 0.93 (95% CI 0.23–3.7) per 1000 person-years for patients without cirrhosis. Overall mortality was significantly higher amongst patients with HCC (4.4% vs. 30.8%, *p* < 0.001). In patients with available liver biopsy (*n* = 249, 25%), advanced fibrosis (F3–F4) was significantly associated with higher HCC incidence, but not steatosis, lobular inflammation, nor ballooning. In the overall cohort, FIB-4 ≥1.3 (HR 8.46, 95% CI 1.06–67.4, *p* = 0.044) and older age (HR 1.06, 95% CI 1.01–1.11, *p* = 0.025) were associated with increasing risk of HCC over time, whereas in cirrhotic patients predictors of HCC included decreasing values of albumin (HR 0.34, 95% CI 0.13–0.87, *p* = 0.024), platelets (HR 0.98, 95% CI 0.98–0.99, *p* = 0.001), and increasing values of liver stiffness (HR 1.03, 95% CI 1.00–1.06, *p* = 0.016). Conclusions: In a Spanish cohort of NAFLD patients, HCC was rare in non-cirrhotic patients. NITs might play a relevant role at predicting HCC.

## 1. Introduction

Liver cancer is considered the sixth-most common cancer and the second-most common cause of cancer-related death worldwide [1]. Hepatocellular carcinoma (HCC) represents 75–85% of all liver cancer and is associated with poor prognosis and higher need for liver transplant [1]. Importantly, non-alcoholic fatty liver disease (NAFLD) is nowadays the fastest increasing underlying cause of HCC globally [2,3,4].

Contrary to other causes of liver disease, there are increasing data suggesting that HCC may develop in the absence of cirrhosis in NAFLD patients [2,5]. Most data come from United States and United Kingdom reports [6,7]. However, these results have not been consistently reported across different geographical settings and, in particular, information in Southern European countries is limited. The increased risk of HCC in non-cirrhotic NAFLD patients implies a challenge for clinicians due to the absence of clear recommendations regardingt surveillance strategies in this scenario [2]. Sanyal et al., recently showed that patients with both fibrosis stages 3 and 4—due to NASH—had an increased risk of HCC development over time compared to those without advanced fibrosis [8].

In clinical practice, NAFLD patients are evaluated thorough non-invasive tests (NITs), such as serologic scores (i.e., FIB-4 index) or vibration-controlled transient elastography (VCTE) for disease staging and risk assessment, with liver biopsy usually being restricted for high-risk patients and consideration for inclusion in clinical trials at liver clinics. Meanwhile, whereas some studies suggest that liver stiffness measurements (LSM) by VCTE may accurately predict the development of HCC in patients with advanced liver disease [9,10,11], it remains unclear whether NITs can be used for HCC development prediction in NAFLD, particularly in the early assessment of patients with undiagnosed advanced liver disease [12]. Interestingly, recent studies found a significantly higher HCC cumulative incidence in NAFLD patients with a FIB-4 index ≥1.3 [13,14,15]. Yet, to date, the diagnosis of HCC risk in patients with chronic liver disease by NITs with LSM and FIB-4 index remains problematic due to its low area under the curve, which is not sufficient to enclose patients with high risk of HCC in actual clinical practice [16]. 

We aimed to analyze the incidence of HCC in an unicentric Spanish cohort of patients with NAFLD and investigate whether NITs are good predictors of HCC development in cirrhotic and non-cirrhotic patients. 

## 2. Materials and Methods

### 2.1. Design and Setting

Single center, retrospective analysis of a prospectively collected cohort. Vall d’Hebron University Hospital is a 1200-bed hospital providing care to a reference population of approximately 650,000 people. From January 2016 onwards, a monographic NASH clinic was created and a prospective collection of data with patients diagnosed with NAFLD was initiated. Data were collected until 31 October 2021. 

### 2.2. Patients

Diagnosis of NAFLD: either more than 5% of hepatocytes with steatosis in liver biopsy or controlled attenuation parameter (CAP) ≥ 275 dB/m by VCTE; diagnosis of HCC was based on imaging techniques (i.e., contrast-enhanced imaging such as multiphase computed tomography or magnetic resonance imaging showing hypervascularity in the arterial phase and a decreased signal compared with the rest of the liver in the venous and/or delayed phases of the study) plus pathology in non-cirrhotic patients or in patients in whom imaging findings were not conclusive [2]; diagnosis of cirrhosis was based in either histology (fibrosis 4 stage in liver biopsy), imaging volumetric alterations, or direct/indirect signs of portal hypertension. Metabolic syndrome was defined according to NCEP-ATPIII criteria [17].

Exclusion criteria: patients with other causes of liver disease and those with engaged in risk-inducing alcohol consumption (20 g daily for females and 30 g daily for males), patients with prior diagnosis of HCC before the inclusion in the prospective cohort, cirrhotic subjects who did not attend a follow-up, or those with a follow-up time <6 months.

### 2.3. Collected Variables

All patients: sex, age, alcohol consumption, smoking, metabolic risk factors (arterial hypertension, type 2 diabetes mellitus, dyslipidemia, overweight/obesity), blood tests (lipid profile, hemoglobin, INR, platelets, transaminases, bilirubin, albumin, glycated hemoglobin, insulin, C peptide), NITs (steatosis, cirrhosis, and portal hypertension signs in liver ultrasound, LSM and CAP in VCTE, FIB-4 index), diagnosis of cirrhosis, time of follow-up, death, and cause of death.

Cirrhotic patients: hepatic venous portal gradient (HVPG), esophageal varices in upper endoscopy, Child–Pugh score, MELD score, hepatic decompensations (upper digestive bleeding, hepatic encephalopathy, ascites), liver transplant. Cirrhotic patients were systematically followed every 6 months with abdominal US and blood tests.

HCC-related: number of lesions, size, modified Barcelona Clinic Liver Cancer (BCLC) staging system score [18] at diagnosis, and type of treatment.

Liver-biopsy-related variables: where dichotomized (yes/no) in patients with available liver biopsy in order to calculate HCC incidence according to histologic findings as follows: steatosis (fat in ≥ 5%hepatocytes), lobular inflammation (≥2 foci/200×), hepatocyte ballooning (grade ≥ 2), advanced fibrosis (F3–F4).

### 2.4. Outcomes

Primary: cumulative incidence of HCC. Only HCC diagnosed from the prospective collection of data in January 2016 were included in the analysis.

Secondary: mortality and hepatic decompensations. 

### 2.5. Statistical Analysis

Continuous variables are expressed in mean (SD) or median (IQR) according to data distribution. Categorical variables are expressed in total number and percentages. Differences between baseline characteristics of HCC and non-HCC patients were tested using the chi-square test for categorical variables and the Wilcoxon rank-sum test for continuous variables. Comparisons between patients with and without HCC and with and without cirrhosis were performed through Chi^2^ test or Kruskal–Wallis test when appropriate. Since not all patients in the cohort had liver biopsy results available, to calculate the area under the receiver operating characteristics curve (AUROC) of NITs (LSM and FIB-4) to predict HCC, we used the LSM cutoff values suggested by Eddowes et al., to classify the estimation of the fibrosis grade from the LSM results [19]. The cutoff values were defined as ≤8.1 kPa for F0–F1 (no or mild fibrosis), ≥8.2 kPa for F ≥ 2 (moderate fibrosis), ≥9.7 kPa for F ≥ 3 (severe fibrosis), and ≥13.6 kPa for F4 (cirrhosis). Kaplan–Meier survival curves analyzing the occurrence of HCC according to the presence of cirrhosis, FIB-4 adjusted to age, LSM, and fibrosis in liver biopsy were built, with categories compared through chi-square log-rank test. For the analysis of risk factors of HCC development, Cox regression analysis that included variables with *p* < 0.10 in the univariate analysis were used. A two-sided *p* < 0.05 was considered to be statistically significant. Missing values were kept as missing, and no specific statistical procedures were used for imputations. The statistical analysis was performed using SPSS for Windows, Version 21.0 (SPSS Inc, Chicago, IL, USA).

## 3. Results

### 3.1. Sample

**Figure 1:**
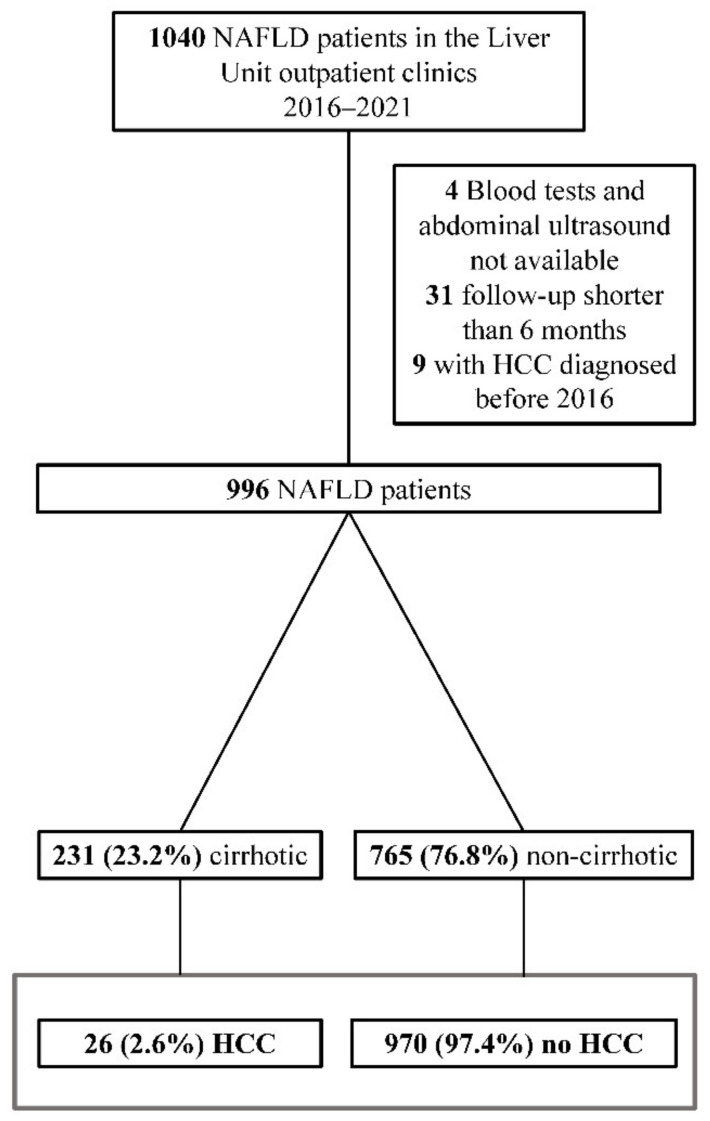
Flowchart of patients’ disposition. HCC: hepatocellular carcinoma.

After excluding patients lacking enough clinical information or follow-up, and those still alive that had already been diagnosed with HCC before the beginning of the study period (*n* = 9), 996 patients were included in the analysis, of whom 26 (2.6%) were diagnosed with HCC during follow-up (Figure 1). Median follow-up time was 2.5 (1.9–3.6) years. 

### 3.2. Baseline and Clinical Characteristics

The overall features of the NAFLD cohort, alongside the characteristics of patients with and without a new diagnosis of HCC during the study period are shown in Table 1. Female sex was present in 52.1% of patients without HCC and only in 13.2% of patients in the HCC group. Patients without HCC were significantly younger. Metabolic syndrome was present in 19.3% (*n* = 193) of all patients, and while there were no differences between the groups without and with HCC regarding mean BMI and rates of arterial hypertension, dyslipidemia was significantly more frequent in the former, whereas T2DM was significantly more frequent in the latter. Two-hundred forty-nine patients (25%) had available liver biopsy (24.5% of the no-HCC patients and 42.3% of HCC patients). Advanced fibrosis (F3–F4) was found in 53.7% of non-HCC and 90.9% of HCC patients. 

Out of the 996 subjects, 231 (23.2%) had cirrhosis at baseline. The characteristics of patients with cirrhosis according to the diagnosis of HCC are shown in Table 2. In the non-HCC group, 207 (21.3%) of patients had a diagnosis of cirrhosis at baseline, whereas 24 (92.3%) did in the HCC group. This was accompanied by significantly lower mean counts of platelets and albumin, and higher levels of bilirubin, as well as FIB-4 index and LSM in the latter group. Moreover, 90.9% and 62.5% of cirrhotic patients were Child–Pugh class A in the non-HCC group and the HCC group, respectively, whereas there were no significant differences between both groups regarding portal hypertension hallmarks.

### 3.3. Incidence and Characteristics of HCC

**Figure 2:**
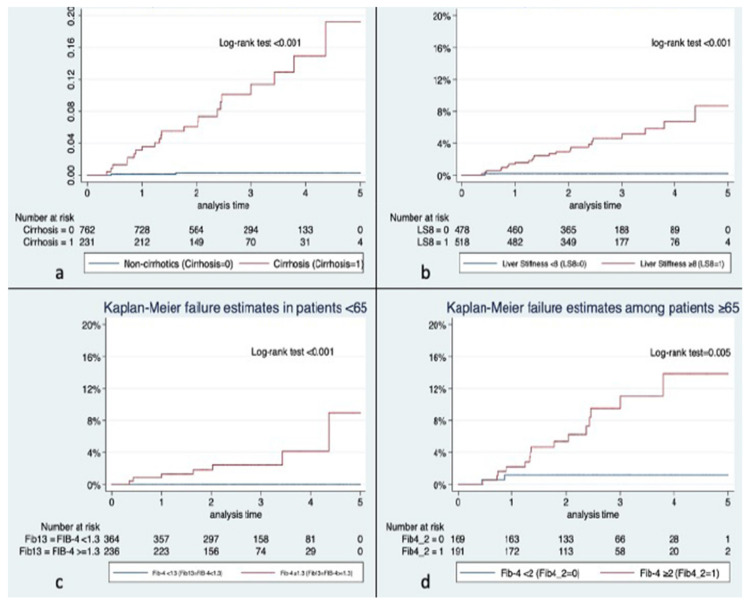
Kaplan–Meier survival curves for hepatocellular carcinoma incidence according to the presence of cirrhosis (**a**), liver stiffness measurement (**b**), and FIB-4 index adjusted to age (**c**,**d**).

Overall HCC incidence rate was 9.49 (95% CI 6.4–13.9) per 1000 person-years. When stratifying patients by cirrhosis status, the incidence rate for non-cirrhotic patients was 0.93 (95% CI 0.23–3.7) per 1000 person-years and 41.2 (95% CI 27.6–61.6) per 1000 person-years for patients with cirrhosis. As shown in Figure 2, the cumulative incidence of HCC was significantly higher in patients with cirrhosis, LSM (>8 kPa), and FIB-4 (≥1.3 in patients < 65 years and ≥2 in patients > 65 years).

**Figure 3:**
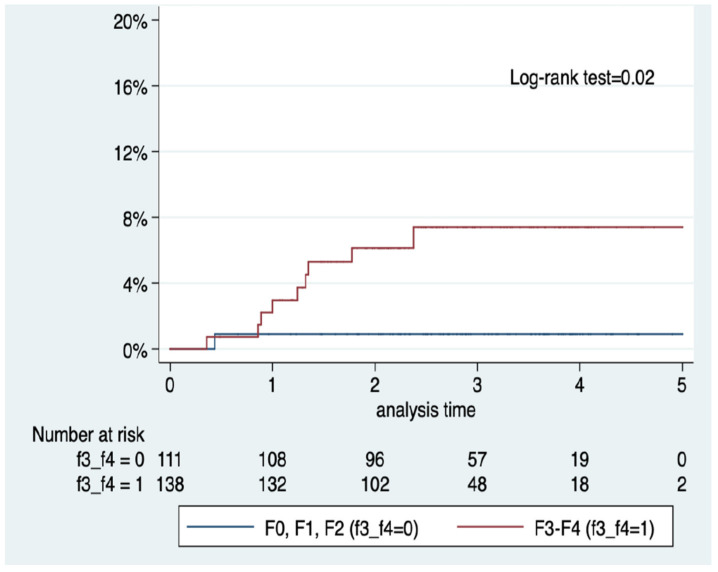
Kaplan–Meier survival curves for hepatocellular carcinoma incidence according to the presence of advanced fibrosis (F3–F4) in liver biopsy (*n* = 249).

Amongst the 249 patients with available liver biopsy, the incidence of HCC was not significantly different between patients with and without steatosis (6/207 (2.9%) vs. 4/40 (10%); *p* = 0.06). Similarly, there were no significant differences in the incidence of HCC when comparing patients with and without significant lobular inflammation (3/51 (5.8%) vs. 6/192 (3.1%); *p* = 0.4). Furthermore, the incidence of HCC was not significantly different between patients with and without prominent ballooning (1/90 (1.1%) vs. 8/153 (5.2%); *p* = 0.1). In contrast, patients with biopsy-proven grade 3–4 fibrosis had a significantly higher incidence of HCC when compared to patients with lower grades of fibrosis (10/138 (7.25%) vs. 1/111 (0.9%); *p* = 0.02). Figure 3 shows the Kaplan–Meier curve (log-rank *p* = 0.02).

Most patients (80.8%, *n* = 21) had a BCLC stage 0 or A at diagnosis and first-line therapy was aimed to be curative in 80.8% (*n* = 21). The median size was 30 (16–35) mm and the majority of patients had a single lesion (*n* = 19). Surgical treatment was applied in 23.1% (*n* = 6), radiofrequency ablation and trans-arterial chemoembolization (TACE) were performed in 10 (38.5%) and 7 (26.9%), respectively. Systemic therapy was used in two patients (Table 3). 

### 3.4. Liver Events and Death

In the non-HCC group, 29.6% of patients with cirrhosis presented hepatic decompensation during follow-up, whereas 71.4% of cirrhotic patients in the HCC group decompensated (*p* < 0.001). The most frequent decompensation in the non-HCC group was ascites (31.1%). Meanwhile, hepatic encephalopathy was the most frequent type of liver event in HCC patients, occurring in 41.6% of them. Liver transplant was significantly more frequent in HCC patients (0.4% vs. 37.5%, Table 2).

Overall mortality during follow-up was 5.1% (51 deaths), with liver failure (*n* = 13) and infections (*n* = 13) being the most common causes, while HCC was the direct cause of death in 2 (4%) patients. Mortality was significantly higher amongst HCC patients in the overall cohort (4.4% vs. 30.8%; *p* < 0.001, Table 1); however, this was not the case amongst cirrhotic patients (17.8% vs. 33.3%; *p* = 0.09, Table 2).

### 3.5. Predictors of HCC

In the overall cohort, multivariable analyses yielded two robust models when introducing NITs (FIB-4 index and LSM) (Table 4). In Model 1, older age and FIB-4 index ≥1.3 were associated with an increased risk adjusted odds of HCC, whereas T2DM and BMI, although entering the model, did not reach statistical significance. Model 2 included the following predictors: platelet count and albumin (the higher the value, the lower the risk), and liver stiffness (increasing values associated with increased risk of HCC over time).

The AUROC (95% CI) of FIB-4 and LSM for HCC diagnosis were 0.87 (0.78–0.96) and 0.85 (0.77–0.93), respectively. Regarding fibrosis stage, the AUROC (95% CI) of F0–F1, F2, F3, and F4 were 0.26 (0.22–0.30), 0.47 (0.43–0.51), 0.45 (0.41–0.48), and 0.80 (0.74–0.87), respectively. In patients ≥65 years, a 2.0 FIB-4 threshold had an AUROC of 0.65 (0.56–0.75). In younger patients (<65 years), a 1.3 FIB-4 threshold had an AUROC of 0.80 (0.78–0.82).

## 4. Discussion

### 4.1. Major Findings

In the present study, conducted in a large cohort of NAFLD patients followed for a median 2.5 years in a Southern European center, there are four major findings. First, the vast majority of HCC developed in patients with cirrhosis at baseline, and therefore the incidence of HCC in non-cirrhotic NAFLD was very low in our cohort. Second, HCC diagnosis is associated to high rates of overall mortality and hepatic decompensation in cirrhotic patients. Third, classical predictors of HCC such as advanced liver fibrosis, and platelets count and albumin in cirrhotic patients were found. Lastly, NITs as FIB-4 and LSM were found to have predictive ability for HCC.

### 4.2. Incidence of HCC

Although the total number of HCC diagnoses in our cohort is relatively small, incidence rates are not lower but rather higher than those of large and well-studied NAFLD cohorts. For instance, in their recent study, Sanyal et al., diagnosed nine cases of HCC amongst 1761 patients with biopsy-confirmed NAFLD, only one of them among F4 patients [8]. Cumulative probability of developing HCC was below 0.5/100 patients-year for F0–2, around 2/100 patients-year for F4, and close to 4/100 patients-year for F3 [8], the latter of which is similar to the overall cumulative incidence in our cohort.

Interestingly, we found that both F3 and F4 (advanced liver fibrosis) were associated with a significantly higher likelihood of developing HCC amongst patients with available liver biopsy, but not amongst the whole cohort. Oppositely to Sanyal et al.’s study and other American and English studies [1,2,3,4,5,6,7,8], the incidence of HCC in non-cirrhotic patients was very low in our cohort, only 7.7% (2/26) of total HCC new diagnoses. In a recent systematic review with metanalysis that analyzed 30 studies encompassing 13,371 subjects with NAFLD-associated HCC, Castellana et al., found that 37% (95% CI 28% to 46%) of patients developed HCC without cirrhosis [7]. In European studies, results were similar, with 36% (95% CI 13% to 58%) of HCC occurring in non-cirrhotic patients [7]. However, it is worth noting that data coming from Southern European countries are scarce. In a multicenter study encompassing Northern Italian hospitals, Piscaglia et al., analyzed 145 patients with NAFLD-associated HCC, and only 50% had cirrhosis [20]. Yet, average alcohol intake in Spain has been higher overall than in Italy and Anglo-Saxon countries during the last three decades [21], and although NAFLD patients in our study do not have either current or recent harmful alcohol consumption, prior exposure to alcohol might act as a contributing factor in carcinogenesis and fibrogenesis in our area, therefore reducing the proportion of non-cirrhotic NAFLD patients with HCC. Additionally, further studies assessing the impact of ethnicity, prevalence of PNPLA3 and TM6SF2 polymorphisms, as well as major epigenetic factors—such as Mediterranean diet and physical exercise—are needed to understand such wide variability.

### 4.3. Decompensations, Liver Transplant, and Mortality

All-cause mortality was 5.1% in our study. In the Sanyal et al., study, with 10-year follow-up, all-cause mortality was 2.7% [8]. In our cohort, 23.2% of patients were cirrhotic at baseline, whereas only 167 were F4 patients at baseline in the Sanyal study [8]. In the latter, there were 39 hepatic decompensations during follow-up (17 of which in F4 patients at baseline), thus 2.2% of the overall cohort and 35.9% of cirrhotics decompensated. Meanwhile, we recorded 74 decompensations amongst 996 patients and 231 cirrhotics (7.4% and 32%) in a much shorter period. Patients with HCC decompensated significantly more frequently in our cohort. Thus, potential explanations to the differing outcomes in Sanyal et al.’s study and our own might encompass, among other thing, the higher rate of cirrhosis at baseline and the higher incidence of HCC.

### 4.4. Predictors of HCC

Notably, T2DM was not found to be a predictor of HCC development amongst patients with NAFLD as widely described in the literature [1,2]. Meanwhile, LSM was found to be a predictive factor of HCC in cirrhotics. This is an interesting finding as VCTE is not recommended as a prediction tool for HCC development in guidelines [12]. Recently, Shearer and colleagues analyzed electronic health records data from 3028 patients in a multistate American study where the natural history of advanced chronic liver disease (LSM > 10 kPa) was assessed by means of VCTE [22]. Remarkably, the cumulative incidence of HCC before decompensation was low (1.3%) for NAFLD patients at 5 years after VCTE, whereas albumin bilirubin (ALBI) score was associated with the development of HCC from the compensated state [22]. Other prediction tools including LSM have been recently published [23,24]. In addition, we also found albumin and platelet count to be associated with an increased risk of HCC, which has already been described in NAFLD [25,26,27]. However, the finding of FIB-4 index ≥1.3 being associated to an increased risk of developing HCC over time has just recently been described, i.e., Loosen et al., found a much higher cumulative risk of HCC in NAFLD patients with FIB-4 ≥ 1.3 (0.47% vs. 0.04%; *p* < 0.001) [13]. Notably, Loosen et al., study was based on a large German outpatient population, whereas ours is based in a hospital-reference cohort with a large proportion of patients with advanced liver disease, which adds to the potential use of NITs in non-population-based studies or in primary care practice but in the liver outpatient clinic or ward.

### 4.5. Limitations

Our study is constrained by several limitations. It is a single center study, and diagnosis of NAFLD was not confirmed via biopsy in all cases. Moreover, VCTE was not systematically performed. There might be a selection bias because only patients in the NAFLD monographic clinic were analyzed, whereas other potential HCC in non-cirrhotic NAFLD patients not followed in our clinic might have been diagnosed in our institution. The time elapsed since the onset of cirrhosis prior to the inclusion in the prospective cohort was unknown for some patients. In spite of these limitations, ours is the first large study providing data on NAFLD-HCC in a Southern European country, and findings—particularly those related to the potential utility of NITs in the prediction of HCC—might be relevant for public policy and to guide further studies in countries where data on NAFLD-HCC are lacking.

## 5. Conclusions

In our cohort, HCC was rare amongst non-cirrhotic patients. In spite of being largely diagnosed at early BCLC stages, HCC was associated with high rates of liver decompensation. Advanced liver fibrosis by biopsy and non-invasive tests (i.e., FIB-4 and LSM) were associated with the risk of developing HCC over time.

## Figures and Tables

**Table 1:** Characteristics and outcomes according to the diagnosis of hepatocellular carcinoma (entire cohort).

	No HCC *n* = 970	HCC *n* = 26	*p*
Female, *n* (%)	505 (52.1)	2 (7.6)	**<0.001**
Age, median (IQR)	60 (51–68)	69 (60–72)	**0.001**
Arterial hypertension, *n* (%)	530 (54.6)	10 (38.5)	0.102
Dyslipidemia, *n* (%)	595 (61.3)	8 (30.8)	**0.002**
Type 2 diabetes mellitus, *n* (%)	396 (40.8)	19 (73.1)	**0.001**
Body mass index, median kg/m^2^ (IQR)	30.9 (27.7–34.7)	29.8 (27.5–35)	0.4
Metabolic syndrome, *n* (%)	187 (19.3)	6 (23)	0.6
Hb1Ac, median g/L (IQR)	5.8 (5.5–6.7)	5.8 (5–6.5)	0.2
Platelets, median × 10^9^/L (IQR)	228 (176–278)	102 (72–139)	**<0.001**
Bilirubin, median mg/dL (IQR)	0.61 (0.49–0.85)	1.1 (0.86–1.83)	**<0.001**
AST, median U/L (IQR)	32 (24–45)	48 (33–57)	**0.002**
ALT, median U/L (IQR)	36 (24–54)	40 (28–45)	0.9
Alkaline phosphatase, median U/L (IQR)	90 (72–115)	125 (96–189)	**<0.001**
GGT, median U/L (IQR)	63 (36–124)	187 (91–323)	**<0.001**
Albumin, median g/L (IQR)	4.3 (4.1–4.5)	3.8 (3.3–4.2)	**<0.001**
Total cholesterol, median mg/dL (IQR)	197 (173–228)	175 (145–200)	**<0.001**
c-HDL median mg/dL (IQR)	48 (42–58)	45 (41–59)	0.7
c-LDL median mg/dL (IQR)	117 (95–142)	100 (85–130)	0.09
Triglycerides median mg/dL (IQR)	137 (100–191)	91 (74–117)	**<0.001**
C Peptide, median U/L (IQR)	2.45 (1.81–3.22)	2.74 (2.23–2.83)	0.6
FIB-4, median (IQR)	1.3 (0.9–2.1)	5.5 (2.6–7.5)	**<0.001**
Liver stiffness, median kPa (IQR)	7.8 (5.4–12.6)	32 (17.8–58.2)	**0.001**
Control attenuation parameter, median dB/m (IQR)	319 (273–359)	297 (250–350)	0.3
Steatosis in abdominal US, *n* (%)	778 (82.8)	8 (30.8)	**<0.001**
Fibrosis in patients with available liver biopsy, *n* (%)	*n* = 238	*n* = 11	**0.009**
F0	29 (12.1)	1 (9.1)	
F1	47 (19.8)	0	
F2	34 (14.3)	0	
F3	43 (18.1)	0	
F4	85 (35.7)	10 (90.9)	
Death, *n* (%)	43 (4.4)	8 (30.8)	**<0.001**
Causes of death			**0.005**
Liver Failure, *n* (%)	12 (28%)	1 (12%)	
HCC, *n* (%)	0	2 (25%)	
Cardiovascular, *n* (%)	6 (14%)	0	
Other neoplasms, *n* (%)	8 (18%)	4 (50%)	
Infectious, *n* (%)	12 (27.9%)	1 (12.5%)	
Other causes, *n* (%)	5 (11.6)	0	
Follow-up, median years (IQR)	2.5 (1.9–3.6)	1.4 (0.85–2.4)	**<0.001**

HCC: hepatocellular carcinoma; IQR: interquartile range; ALT: alanine aminotransferase; AST: aspartate aminotransferase; GGT: gamma glutamyl transferase; Hb1Ac: glycosylated hemoglobin; HVPG: hepatic venous pressure gradient; US: ultrasound.

**Table 2:** Characteristics and outcomes of cirrhotic patients according to the diagnosis of hepatocellular carcinoma.

	No HCC *n* = 207	HCC *n* = 24	*p*
% over total cohort	21.3	92.3	**<0.001**
Female, *n* (%)	98 (47.3)	2 (8.3)	**<0.001**
Median age, years (IQR)	65 (58–73)	69 (60–71)	0.3
Arterial hypertension, *n* (%)	145 (70)	10 (41.6)	**0.01**
Dyslipidemia, *n* (%)	122 (58.9)	8 (33.3)	**0.02**
T2DM, *n* (%)	148 (71.5)	17 (70.8)	0.9
HbA1c, %	6.2 (5.5–7.3)	5.8 (5–6.5)	0.07
BMI, kg/m^2^	31.9 (28.5–35.2)	30 (27.9–35.1)	0.2
Metabolic syndrome, *n* (%)	70 (33.8)	6 (25)	0.3
Platelets, ×10^9^/L, median (IQR)	139 (94–208)	99.5 (71.5–126)	**0.01**
Bilirubin, median mg/dL (IQR)	0.8 (0.6–1.1)	1.2 (0.8–1.9)	**<0.001**
AST, median IU/L (IQR)	43 (30–57)	51 (33.5–58.5)	0.3
ALT, median IU/L (IQR)	36 (22–58)	40 (28.5–45)	0.7
Alkaline phosphatase, median IU/L (IQR)	103 (76–137)	129 (98–191)	**0.02**
GGT, median UI/L (IQR)	102 (55–223)	171 (87–326)	**0.01**
Albumin, median UI/L (IQR)	4.1 (3.8–4.4)	3.7 (3.2–4.1)	**0.001**
FIB-4, median (IQR)	3.3 (1.9–5.2)	6.09 (3.1–7.8)	0.054
Steatosis in US, *n* (%)	116 (56.5)	7 (29.1)	**0.01**
CAP, median dB/m (IQR)	310.5 (262–360)	308 (242.5–363.5)	0.8
Liver stiffness, median kPa (IQR)	27.3 (17.9)	38.3 (22.3)	**0.031**
Liver stiffness > 15 kPa, *n* (%)	137 (73.6%)	11 (84.6%)	0.3
Median HVPG, mmHg (IQR) *	10.5 (6.5–13)	8 (8–8)	0.5
Portal hypertension signs in abdominal US, *n* (%)	115 (56.1)	18 (78.2)	**0.04**
Varices, *n* (%) **	75 (45.1)	14 (60.8)	0.18
Child score, *n* (%)			**0.001**
A	157 (90.8)	15 (62.5)	
B	15 (8.6)	8 (33.3)	
C	1 (0.5)	1 (4.1)	
Child–Pugh score ≥6, *n* (%)	37 (17.8)	13 (54.1)	**<0.001**
MELD	7.7 (6.7–9.4)	9.4 (7.5–12)	**0.004**
Hepatic decompensation during follow-up, *n* (%)	59 (29.6)	15 (71.4)	**<0.001**
Ascites, *n* (%)	48 (31.1)	9 (37.5)	0.5
Hepatic encephalopathy, *n* (%)	26 (16.8)	10 (41.6)	**0.005**
Upper digestive bleeding, *n* (%)	22 (14.2)	5 (20.8)	0.4
Liver transplant, *n* (%)	1 (0.4)	9 (37.5)	**<0.001**
Death	37 (17.8)	8 (33.3)	0.09
Median follow-up, years (IQR)	2.2 (1.8–3.5)	1.5 (0.8–2.7)	**0.02**

* HVPG available in 41 patients; ** Gastroscopy available in 186 patients. ALT: alanine aminotransferase; AST: aspartate aminotransferase; GGT: gamma glutamyl transferase; Hb1Ac: glycosylated hemoglobin; HVPG: hepatic venous pressure gradient; US: ultrasound.

**Table 3:** Characteristics of hepatocellular carcinoma cases included in the analysis.

Total Number of HCC	26 (100)
HCC BCLC stage, *n* (%)	0: 6 (23)
	A: 15 (57.6)
	B: 2 (7.6)
	C: 1 (3.8)
	D: 1 (3.8)
	Not classified 1 (3.8)
HCC size, median (IQR)	30 (16–35)
HCC type of treatment, *n* (%)	Curative: 21 (80.7)
	Surgical 6 (23)
	TACE: 7 (27)
	Radiofrequency Ablation: 10 (38.4)
	Systemic 2 (7.6)
HCC relapses, *n* (%)	8 (30.7)

**Table 4:** Summary of the multivariable analyses of factors associated with the incidence of hepatocellular carcinoma in NAFLD patients.

	HR	95% Confidence Interval		*p*
**Model 1 (overall cohort)**				
Type 2 diabetes mellitus	1.51	0.58	3.89	0.394
Body mass index (×1 kg/m^2^)	0.99	0.91	1.09	0.930
Age (×1 year)	**1.06**	**1.01**	**1.11**	**0.025**
FIB-4 ≥ 1.3	**8.46**	**1.06**	**67.37**	**0.044**
**Model 2 (cirrhotics)**				
Platelets (x + 10 × 10^9^/L)	**0.98**	**0.98**	**0.99**	**0.001**
Albumin (x + 1 IU/L)	**0.34**	**0.13**	**0.87**	**0.024**
Liver stiffness (x + 1 kPa)	**1.03**	**1.00**	**1.06**	**0.016**

## Data Availability

Data will be available upon request.
